# Hydrogen-rich solution attenuates cold ischemia-reperfusion injury in rat liver transplantation

**DOI:** 10.1186/s12876-019-0939-7

**Published:** 2019-02-08

**Authors:** Keiichi Uto, Seisuke Sakamoto, Weitao Que, Keita Shimata, Shintaro Hashimoto, Masataka Sakisaka, Yasuko Narita, Daiki Yoshii, Lin Zhong, Yoshihiro Komohara, Xiao-Kang Li, Yukihiro Inomata, Taizo Hibi

**Affiliations:** 1 Department of Transplantation and Pediatric Surgery, Postgraduate School of Medical Sciences, Kumamoto University, 1-1-1 Honjo, Kumamoto, 860-8556 Japan; 2 Organ Transplant Center, National Center for Child Health and Development, 2-10-1 Okura, Setagaya-ku, Tokyo, 157-8535 Japan; 3 Division of Transplantation Immunology, National Research Institute for Child Health and Development, 2-10-1 Okura, Setagaya-ku, Tokyo, 157-8535 Japan; 4 Department of Surgery, Shanghai General Hospital, Shanghai Jiao Tong University School of Medicine, 800 Dongchuan RD. Minhang District, Shanghai, 201100 China; 5 Department of Cell Pathology, Graduate School of Medical Sciences, Kumamoto University, 1-1-1 Honjo, Kumamoto, 860-8556 Japan

**Keywords:** Hydrogen, Liver transplantation, Ischemia-reperfusion injury, Heme oxygenase-1, Rat

## Abstract

**Background:**

Liver transplantation (LT) is considered the standard treatment for end-stage liver disease, but ideal donors remain in limited supply, resulting in an unavoidable increase in the need to use grafts from marginal donors. The attenuation of ischemia-reperfusion injury (IRI) in such marginal donors is therefore crucial for reducing the possibility of the primary non-function of grafts and graft loss. Some reports have found that molecular-hydrogen showed antioxidant and anti-inflammatory effects in preventing IRI in some non-hepatic transplant models. Therefore, we investigated whether or not molecular-hydrogen could attenuate IRI in LT model rats.

**Methods:**

We used a hydrogen-rich water bath to dissolve hydrogen into solution and graft tissues and performed isogenic and orthotopic LT in Lewis rats with University of Wisconsin (UW) solution. Blood and tissue samples were collected 6 h after the reperfusion. Hepatic enzymes in serum were measured. Pathological findings including the expressions of cytokines and heme oxygenase (HO)-1 in liver tissues were evaluated.

**Results:**

The concentration of hydrogen inside the graft tissues increased depending on the storage time, plateauing after 1 h. Serum liver enzyme levels were significantly lower and the histology score of liver damage markedly attenuated in the group given grafts preserved in hydrogen-rich UW solution than in the control group. The hydrogen-rich UW solution group also showed less oxidative damage and hepatocyte apoptosis than the control group, and the expression of proinflammatory cytokines tended to be lower while the protein levels of HO-1 were significantly increased (*n* = 3–12 per group, *P* < 0.05).

**Conclusions:**

Storage of liver grafts in hydrogen-rich UW solution resulted in superior functional and morphologic protection against IRI via the up-regulation of HO-1 expression.

**Electronic supplementary material:**

The online version of this article (10.1186/s12876-019-0939-7) contains supplementary material, which is available to authorized users.

## Background

Liver transplantation (LT) is considered the standard treatment for end-stage liver disease [1]. However, although the innovation of immunosuppression therapies, perioperative management and surgical procedures and materials, including preservation solutions, have improved the outcomes of LT, the primary non-function of a graft and graft loss still occur at reported rates of 3.8% [2] to 5.8% [3]. Ischemic-reperfusion injury (IRI) has been suggested as a risk factor for adverse events that happen perioperatively [4].

In addition, donor shortage remains an issue, and grafts from marginal donors, including older donors, steatotic donors and donors after cardiac death, are being used increasingly to meet the demand [5]. Because of the vulnerability of such marginal donors to IRI, reducing the risk of IRI is crucial for preventing the primary non-function of grafts and graft loss [6]. Some animal studies have attempted to identify agents that prevent IRI, but most have failed to find a foothold in the clinical routine [7, 8].

However, several recent reports have found that molecular-hydrogen showed antioxidant and anti-inflammatory effects for preventing IRI in some non-hepatic transplant models. The inhalation of hydrogen gas attenuated cerebral IRI, and the preservation of the graft tissue in hydrogen-rich solution was effective in preventing against IRI in rat intestinal, kidney and cardiac transplantation models [9–12].

In the present study, we investigated whether or not a hydrogen-rich solution can attenuate IRI in the setting of orthotopic liver transplantation using a rat model.

## Methods

### Preparation and maintenance of the hydrogen-rich solution

We used a hydrogen-rich water bath (MiZ Co., Ltd., Kanagawa, Japan) to dissolve hydrogen into the liver graft. It consisted of a thermostatic water bath and hydrogen generator that electrolyzed the water. The bath and electrolyzer were connected to a pump, and the water was continuously circulated between the bath and the generator, maintaining the saturated hydrogen concentration and keeping the temperature at 4 °C [13].

University of Wisconsin (UW) solution (ViaSpan; Bristol-Myers Squibb Pharmaceuticals, County Dublin, Ireland) was used as the solvent medium. To make the hydrogen-rich preservation solution, the UW bag was immersed in the hydrogen-rich water bath for more than 24 h, and hydrogen was dissolved into the UW solution through the plastic bag and existed in the target concentration without contamination.

**Fig. 1:**
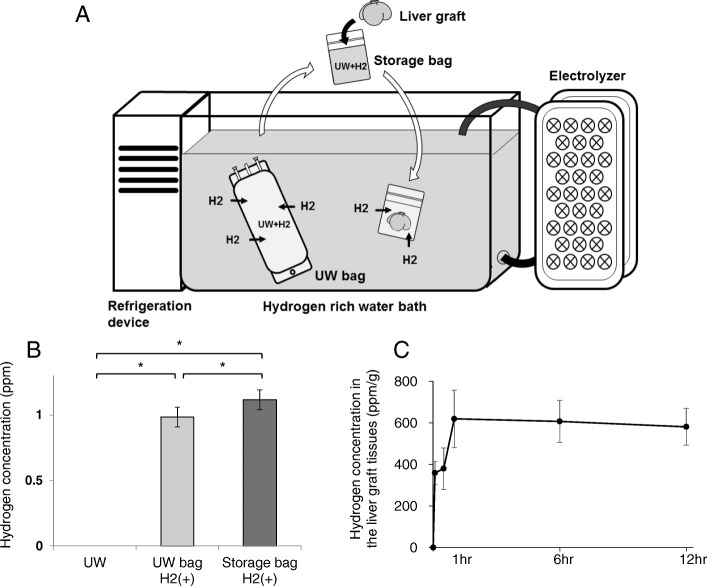
The preparation of hydrogen-rich UW solution and the measurement of the concentration of hydrogen in the UW solution and liver tissue. **a** The cold-water bath and electrolyzer. **b** A significant increase in the hydrogen concentration of the UW solution was noted. UW bag H2 (+): concentration of hydrogen in the UW solution, prepared in the UW bag for more than 24 h. Storage bag H2 (+): concentration of hydrogen in the UW solution, prepared with the liver graft in the plastic storage bag after 12 h of cold storage. (*n* = 6 for each group); *P < 0.05. **c** The hydrogen concentration of the graft tissue increased depending on the storage time, eventually plateauing (*n* = 3 each time)

In the hydrogen therapy group, a liver graft was put in a plastic storage bag filled and sealed with 70 ml cold hydrogen-rich UW solution after the back-table procedure. The storage bag was then immersed in the bath to maintain the concentration of hydrogen (Fig. 1a).

### Measurement of the hydrogen concentration in the solution and liver tissues

The concentration of hydrogen in the solution was measured twice: just before placing the liver graft in the fluid and at the time of implantation. The concentration of hydrogen in the UW solution was measured using a methylene blue-platinum colloid reagent kit (MiZ Co., Ltd.) [14]. We measured the hydrogen in the liver tissues using the liberated gas from the specimen, as previously described [15]. In brief, the tissue samples were homogenized in air-tight tubes using the gentleMACS Octo Dissociator (Miltenyi Biotec GmbH, Bergisch Gladbach, Germany), and the released hydrogen was measured using sensor gas chromatography (SGHA-P1; FIS Co., Ltd., Hyogo, Japan). The concentration of hydrogen was calculated to ppm per 1 g of liver tissue.

### Animals

Inbred male Lewis rats, weighing 220–300 g, were purchased from Charles River Laboratories International, Inc. (Kanagawa, Japan) and SLC Japan (Hamamatsu, Japan). All rats were maintained in a specific-pathogen-free environment and housed under a 12-h dark–light cycle (light from 07:00 to 19:00) at 22 ± 1 °C. They were given free access to standard diet and water and were not fasted before the experiments.

### Orthotopic LT

All of the transplant procedures in this study were performed under a stereo microscope. All surgical procedures were performed with clean but nonsterile instruments, and antibiotics were not used. The body weights of the donor and recipient rats were coordinated to be within roughly the same ranges. Rats were anesthetized by inhalation of 0.8–3% isoflurane (Intervet K.K, Tokyo, Japan) and placed on a warm panel during the surgical procedures and sampling of blood or tissues. Orthotopic LT without arterial reconstruction was performed according to Kamada’s two-cuff method [16] with a minor modification [17, 18]. In brief, at the donor operation, the left phrenic, right suprarenal, right renal veins and branches of the portal veins were ligated and divided. The common bile duct was cut, and a 24-gauge plastic cannula was inserted and fixed as a stent tube. After the injection of 2 mL of lactate Ringer’s solution (Solulact; Terumo, Tokyo, Japan) containing 300 units of heparin (Heparin Na; Fuji Pharma Co., Ltd., Tokyo, Japan) via the penile vein, the hepatic artery was ligated and divided. Before the graft retrieval, the liver was flushed via the portal vein with 10 ml of cold (4 °C) lactate Ringer’s solution, followed by 10 ml of cold normal UW solution at a pressure of 10 cmH_2_O. After perfusion, the graft liver was retrieved and placed in a steel cup on crushed ice with normal UW solution and at the end of the experiment, animals were sacrificed by inferior vena cava incision under deep isoflurane anesthesia. In the back-table procedure, plastic cuffs were attached to the portal vein and infra hepatic vena cava of the graft, and the supra hepatic inferior vena cava was trimmed for the anastomosis. After the back-table procedure, the graft was immediately put in the plastic bag with normal or hydrogen-rich UW solution and maintained at 4 °C for preservation for 12 h. In the recipient operation, the left phrenic and right suprarenal veins and the hepatic artery were ligated and divided. The hepatic duct was cut at the bifurcation level. After clamping the portal vein, the supra hepatic vena cava with the diaphragm and infra hepatic vena cava, the recipient’s liver was taken out. After rinsing it with 10 ml of cold lactate Ringer’s solution, the graft was placed into the recipient abdominal cavity in the orthotopic manner. The supra hepatic vena cava was anastomosed with continuous 7–0 monofilament sutures (PROLENE; Ethicon, Inc., Somerville, MA, USA). After the portal vein connection was made using the cuff technique, the clamps were released, and the allograft was re-perfused. Immediately after infra hepatic vena cava reconstruction with the cuff technique, 0.5 mEq of bicarbonate (8.4% MEYLON; Otsuka Pharmaceutical Factory, Inc., Tokushima, Japan) was injected via the penile vein. Anastomosis of the bile duct was performed by leading and fixing the stent tube into the recipient’s bile duct. In all cases, the anhepatic time was less than 20 min, and the vena cava clamping time was less than 30 min.

### Experimental design

The rats were randomly divided into four groups: no operation (Naïve group, *n* = 3); LT with no graft preservation, simply perfused with normal UW solution (NP group, *n* = 4); LT with 12 h of preservation in normal UW solution (UW group, *n* = 6) and LT with 12 h of preservation in hydrogen-rich UW solution (UW + H2 group, *n* = 12). In the NP group, the recipient operations were continuously performed after donor operation and back-table procedures, and the cold ischemic time was approximately 1 h.

### Serum enzymes analyses

Under a reanesthetized condition, blood and liver tissue samples were collected once at 6 h after reperfusion. Blood was collected via the inferior vena cava and centrifuged to isolate the serum, and at the end of the experiment, animals were sacrificed by inferior vena cava incision under deep isoflurane anesthesia. Serum aspartate aminotransferase (AST), alanine aminotransferase (ALT) and lactic dehydrogenase (LDH) were measured using a Dri-Chem FDC-3500 (FUJIFILM Medical Co., Ltd., Tokyo, Japan).

### Morphologic analyses

Liver tissues excised from the right lobe were fixed in 10% buffered formalin and then embedded in paraffin after dehydration. Liver sections were stained with hematoxylin-eosin. The pathological findings were blindly examined by a pathologist (Y.K.), and the severity of IRI was graded according to the modified Suzuki’s criteria [19]. In terms of the scoring system, three micro-sections, consisting of sinusoidal congestion, hepatocyte vacuolization and leukocyte infiltration, were evaluated in 3 random fields on each slide at × 200 magnification and scored from 0 (none) to 4 (severe), and then the total mean scores in each section were calculated. Immunohistochemical staining was performed to evaluate infiltration of neutrophils and macrophages. Neutrophils were stained using a Naphthol AS-D chloroacetate Esterase staining kit (Muto Pure Chemical, Tokyo, Japan) according to the manufacturer’s protocols, and the positive nuclei were counted in 3 random fields in each group at × 200 magnification.

For the evaluation of macrophage infiltration, ED-1 positive cells were counted using an Anti-CD68, Mouse-Mono (ED1) antibody (Bio-Rad, Oxford, UK). ED-1 is the antigen expressed by most of tissue macrophages and weakly by peripheral blood granulocytes. For the analysis of apoptosis, terminal deoxynucleotidyl transferase-mediated deoxyuridine triphosphate nick end-labeling (TUNEL) staining was performed using an in situ Apoptosis Detection Kit (Wako Pure Chemical, Tokyo, Japan) according to the manufacturer’s protocols, and the positive nuclei were counted.

### Quantitative real-time reverse-transcriptase polymerase chain reaction of injury-related mRNA

Total RNA was isolated from the liver tissues using RNAlater stabilization solution (Life Technologies, Carlsbad, CA, USA) for freezing and ISOGEN (NipponGene, Tokyo, Japan) to extract total RNA. The RNA was then reverse-transcribed to complementary DNA using a PrimeScript RT Reagent Kit (Takara Bio, Shiga, Japan). Messenger RNA (mRNA) levels were quantified by real-time reverse-transcriptase polymerase chain reaction (RT-PCR) using TaqMan on an Applied Biosystems 7900HT sequence detection system (Applied Biosystems, Foster City, CA, USA). The following TaqMan probes and primers were used: chemokine (C-C motif) ligand 2 (CCL2): forward, 5’-TTgCTgCCTgTAgCATCCAC-3′, reverse, 5’-TCATCTTgCCAgTgAATgAgTAgC-3′, probe, 5’-TgTCTCAgCCAgATgCAgTTAATgCCC-3′; high mobility group box chromosomal protein (HMGB)-1: forward, 5’-TggAAgAgAgCTTTTgTCCACA-3′, reverse, 5’-TTgATCACTCCTTgCTTTgCC-3′, probe, 5’-CCCTgCCATTgTggTAgggTAACATTTTC-3′; heme oxygenase (HO)-1: forward, 5’-CAgggTgACAgAAgAggCTAAgAC-3′, reverse, 5’-TCTTTgTgTTCCTCTgTCAgCAgT-3′, probe, 5’-TCCTgCTCAACATTgAgCTgTTTgAggA-3′; SIRT-1 (sirtuin-1): forward, 5’-CAGCATCTTGCCTGATTTGTAAATAC-3′, reverse, 5’-CACCGAGGAACTACCTGATTAAAAA-3′, probe, 5’-TCTCCACGAACAGCTTCACAATCAACT-3′; 18S ribosomal RNA: forward, 5’-ATgAgTCCACTTTAAATCCTTTAACgA-3′, reverse, 5’-CTTTAATATACgCTATTggAgCTggAA-3′, probe, 5’-ATCCATTggAgggCAAgTCTggTgC-3′. The other TaqMan probes and primers, including intercellular adhesion molecule (ICAM)-1, vascular cell adhesion molecule (VCAM)-1, were purchased from Applied Biosystems. Data are expressed as the comparative cycle threshold. The normalized cycle threshold value of each gene was obtained by subtracting the cycle threshold obtained for 18S ribosomal RNA.

### Western blotting analysis

A Western blotting analysis was performed on 30 μg of whole-cell protein from liver tissues. After non-specific binding was blocked, the primary antibodies, anti-HO-1 (ab82585, Abcam, Cambridge, UK) and anti-β-actin (13E5) rabbit mAb (Cell Signaling Technology Inc., Danvers, MA, USA), were incubated. Thereafter, the proteins were visualized and analyzed using β-actin as the protein loading control.

### Oxidative damage measurements

Malondialdehyde (MDA) and 8-hydroxy-2-deoxyguanosine (8-OHdG) levels were measured as oxidative stress markers. Serum and liver tissue MDA levels were assessed using a Lab Assay TBARS kit (Cayman Chemical, Greensboro, MI, USA) according to the manufacturer’s protocols. The serum 8-OHdG levels were measured using an enzyme-linked immunosorbent assay kit (Japan Institute for the Control of Aging, Shizuoka, Japan) according to the manufacturer’s protocols. Tissue immunohistochemical staining of 4-hydoxy-nonenal (ab46545; Abcam) was performed to evaluate the severity of oxidative stress, and the positive nuclei were counted in 3 random fields in each group.

### Statistical analyses

All statistical analyses were performed using the statistical package of PASW Statistics 18 for Windows (IBM, Tokyo, Japan). All results are expressed as the means ± standard deviation. Statistical analyses were performed using Tukey’s test for parametric multiple comparisons. *P* < 0.05 was considered to be significant (Additional file 1).

## Results

### Hydrogen levels in the cold preservation solution and liver tissues stored in hydrogen-rich water bath

Figure 1b shows the hydrogen concentration of the UW solution prepared in the hydrogen-rich water bath. There was no decrease in the hydrogen concentration even after 12 h of preservation the graft liver with a plastic storage bag. The hydrogen concentration of the liver tissues increased depending on the storage time in the bath during the first hour, subsequently plateauing around 600 ppm/g (Fig. 1c).

### Hydrogen-rich UW reduced the liver injury

**Fig. 2:**
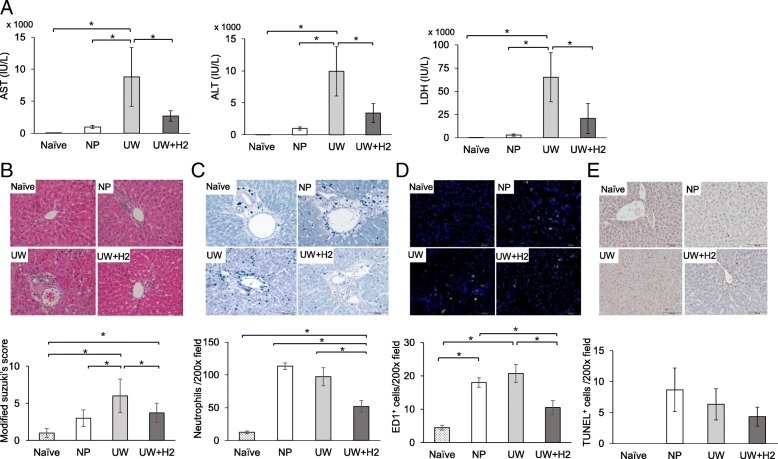
Serum AST, ALT and LDH levels and histological examinations of hepatic injury at 6 h after reperfusion. **a** The increased enzymes levels in the UW preservation group were significantly suppressed in the UW + H2 preservation group (*n* = 3–12 per group). **b** Hematoxylin-eosin staining of liver tissues. The ischemia-reperfusion injury scores of the UW + H2 group were significantly lower (× 100 magnification. *n* = 12–18 per group). **c**, **d** Neutrophil infiltration and ED-1-positive macrophage infiltration were significantly decreased in the UW + H2 group (× 200 magnification. *n* = 3 for each group). **e** TUNEL-positive cells tended to be fewer in the UW + H2 group (× 200 magnification. *n* = 3 for each group); *P < 0.05

Figure 2a shows that the AST, ALT and LDH levels of the UW group were significantly higher than those in the naïve and NP groups. Furthermore, those levels in the UW + H2 group were significantly lower than in the UW group. In the histological assessment of hematoxylin-eosin staining, the UW group showed multiple hemorrhages, dissociation of the cords of hepatocytes and cell ballooning, although those findings were suppressed in the UW + H2 group. Necrosis was rarely seen in any group. The IRI score of the UW + H2 group was significantly lower than that in the UW group and not markedly different from that in the NP group (Fig. 2b).

### Hydrogen-rich UW reduced inflammation and hepatocyte apoptosis in liver tissue

On immunohistochemical staining, massive neutrophils infiltration was detected in the UW group; however, this finding was significantly decreased in the UW + H2 group (Fig. 2c). Furthermore, ED-1 positive macrophage infiltration was significantly less frequent in the UW + H2 group than in the NP and UW groups (Fig. 2d). TUNEL-positive cells tended to be fewer in the UW + H2 group than in the NP and UW groups (Fig. 2e).

### Hydrogen-rich UW reduced injury-related mRNA and protein expression in liver tissue

**Fig. 3:**
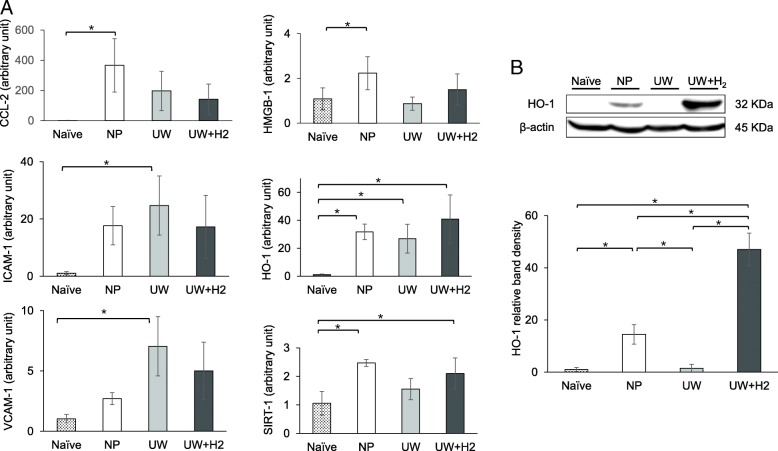
Quantitative real-time reverse-transcriptase polymerase chain reaction for proinflammatory cytokines and chemokines of liver tissues at 6 h after reperfusion. **a** The mRNA levels of CCL2, ICAM-1 and VCAM-1 tended to be lower in the UW + H2 group than in the UW group. The HO-1 and SIRT-1 levels were increased in the UW + H2 group (*n* = 3–5 per group). **b** A Western blot analysis was performed. In the UW + H2 group, the HO-1 protein level was markedly upregulated (*n* = 3 for each group); *P < 0.05

In the measurement of the mRNA levels of proinflammatory cytokines in liver tissues using RT-PCR, the CCL2, ICAM-1 and VCAM-1 levels tended to be lower in the UW + H2 group than in the UW group (Fig. 3a). Similarly, the HO-1 and SIRT-1 levels increased in the UW + H2 group. In the Western blotting analysis, the HO-1 protein level of the liver tissue was markedly upregulated in the UW + H2 group compared with the other groups (Fig. 3b).

### Hydrogen-rich UW suppressed the oxidative stress

**Fig. 4:**
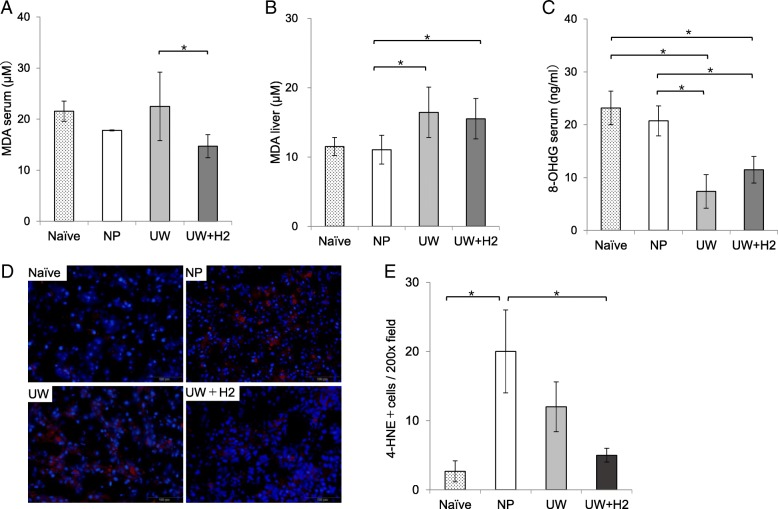
Oxidative damage. **a** The serum MDA levels of the UW + H2 group were significantly lower. **b**, **c** There were no remarkable changes in the MDA levels in liver tissues and the serum 8-OHdG levels. **d**, **e** The number of 4-hydoxy-nonenal-positive cells tended to be lower in the UW + H2 group(× 200 magnification. *n* = 3–6 per group); *P < 0.05

The serum MDA levels in the UW + H2 group were significantly lower than in the UW group (Fig. 4a). The number of 4-hydoxy-nonenal-positive cells tended to be lower in the UW + H2 group (Fig. 4d, e).

## Discussion

This study investigated the therapeutic effects of molecular-hydrogen on IRI in a rat LT model using a hydrogen-rich water bath. We successfully prepared liver grafts with a sufficient concentration of hydrogen during a 12-h preservation period in a cold hydrogen-rich solution. Even with 12-h preservation, hydrogen was still able to attenuate liver injury effectively, and inflammatory changes were unremarkable at 6 h after reperfusion in the serum and histologic analysis. Furthermore, the oxidative liver damage was reduced, and the HO-1 expression was increased in the hydrogen treatment group compared with other groups.

During the ischemic period, the reduced oxygen supply leads to a lack of regeneration of adenosine monophosphate (AMP) to adenosine triphosphate (ATP). Subsequent failure of the ATP-dependent sodium/potassium membrane pump and the accumulation of intracellular sodium lead to the swelling of hepatocytes, and the accumulated AMP is metabolized to hypoxanthine. Although hypoxanthine is normally converted to urea by xanthine dehydrogenase, xanthine dehydrogenase is converted to xanthine oxidase in the ischemic state, and hypoxanthine is therefore not metabolized. After graft reoxygenation, the increased levels of hypoxanthine are converted to xanthine with reactive oxygen species (ROS), such as superoxide, hydrogen peroxide and hydroxyl radical [20]. Those ROS activate adhesion molecules, such as ICAM-1 and VCAM-1, of hepatocytes and sinusoidal endothelial cells, and then adhesion of neutrophils and platelets with cell-swelling lead to a reduction in the microcirculatory blood flow. The migrated neutrophils across the endothelium into the parenchyma reflect the direct damage of hepatocytes and induce ROS production [21, 22]. ROS then activates circulating neutrophils, CD4^+^ T lymphocytes and sinusoidal endothelial cells, leading to the expression of cell-surface adhesion molecules [23, 24]. Because this increase in ROS induces IRI, reducing ROS enhancement is deemed the most effective strategy for ameliorating or preventing IRI.

The first report of a molecular-hydrogen therapeutic effect on IRI was derived from a study on focal ischemia-reperfusion in a model of rat brain [9]. Previous studies in organ transplantation models have shown that hydrogen promotes a therapeutic effect on IRI by reducing oxidative stress, which is led by the chain reaction of ROS [10, 11]. Another study of hydrogen-rich eye drops demonstrated the direct suppression of hydroxyl radical levels in rat retinas in an IRI model [25]. Our study in a rat LT model showed that the UW + H2 group had attenuated liver injury according to a biochemical analysis. Furthermore, in the histological analysis, a lower IRI classification score reflected the suppression of pro-inflammatory molecules and subsequent leukocyte migration and infiltration. Measurements by using serum MDA levels, a marker of lipid peroxidation, were significantly decreased in the hydrogen group. These results suggest that molecular-hydrogen suppressed the inflammatory changes and oxidative stress underlying IRI in LT.

Our study also showed the marked overexpression of HO-1 protein in the liver tissues at 6 h after reperfusion in the UW + H2 group. HO-1 is a component related to heat-shock protein and is a rate-limiting enzyme that catabolizes heme to biliverdin, carbon monoxide and free iron [26]. HO-1 was recently reported to have anti-inflammatory and antioxidant behaviors that prevent organs from developing IRI, including LT models. Previous studies regarding HO-1 inducers in a rat isogenic LT model have suggested that HO-1 also has an antioxidant function, microcirculation maintenance function, modulatory function in the cell cycle, and anti-inflammatory function [27]. A relationship between the overexpression of HO-1 and the attenuation of IRI has been reported in the literature based on findings in some IRI models [10, 28, 29]. Hydrogen-rich solution may upregulate the HO-1 levels in the graft tissues and lead to the protection of livers from IRI.

Hydrogen is a flammable gas; therefore, dissolving it in a solution is safer with regard to management than its gaseous form. In our study, we were able to maintain the concentration of hydrogen in the UW solution and liver tissue over 12 h of cold ischemic time. These findings showed the effectiveness of this storage system for maintaining the hydrogen concentration of the graft tissue in the hydrogen-rich UW solution. It is also worth mentioning that preservation in a hydrogen-rich water bath is a calmer method than high-pressure storage or exposure of the liver graft to bubbling hydrogen for maintaining the hydrogen concentration [11]. Furthermore, the medium can be kept sterile while resolving the liver graft using a hydrogen-rich water bath and infusing through a sealed storage bag. However, a hydrogen-rich water bath is not a readily available system and is not very portable, limitations that have been recognized as important issues to consider in the future. Hydrogen-rich solution may be useful for improving patient outcomes in LT, although studies in large sample size and large animal models are needed to evaluate the effectiveness for the clinical application.

## Conclusions

Sufficient hydrogen distribution in the liver graft was obtained using a hydrogen-rich water bath. The storage of the liver grafts in hydrogen-rich UW solution resulted in superior functional and morphologic protection of the grafts against IRI. The up-regulation of HO-1 was suggested as a mechanism underlying this effect. Our present study showed that the hydrogen-rich solution was able to decrease the oxidative stress and inflammatory changes induced by IRI in a rat LT model.

## Additional file

Additional file 1: Statistical analyses (Tukey's test). (PDF 75 kb)

## Abbreviations

8-OHdG: 8-hydroxy-2-deoxyguanosine
ALT: Alanine aminotransferase
AMP: Adenosine monophosphate
AST: Serum aspartate aminotransferase
ATP: Adenosine triphosphate
CCL2: Chemokine (C-C motif) ligand 2
HO: Heme oxygenase
ICAM: Intercellular adhesion molecule
IRI: Ischemia-reperfusion injury
LDH: Lactic dehydrogenase
LT: Liver transplantation
MDA: Malondialdehyde
mRNA: Messenger RNA
ROS: Reactive oxygen species
RT-PCR: Real-time reverse-transcriptase polymerase chain reaction
TUNEL: Terminal deoxynucleotudyl transferase-mediated deoxyuridine triphosphate nick end-labeling
UW: University of Wisconsin
VCAM: Vascular cell adhesion molecule
