# Supplementary material for: Hydrogen-rich solution attenuates cold ischemia-reperfusion injury in rat liver transplantation
Source: BMC Gastroenterol. 2019 Feb 8;19:25. doi: 10.1186/s12876-019-0939-7 (PMC6368804; doi:10.1186/s12876-019-0939-7)
Supplement: Supplementary file 1 — Statistical analyses (Tukey's test). (PDF 75 kb) [file 12876_2019_939_MOESM1_ESM.pdf]

Statistical analyses (Tukey's test)

			n	P
H2 concentration	UW bag H2	UW	6	0.0000
		UW storage	6	0.0040
	UW storage bag H2	UW	6	0.0000
		UW bag	6	0.0040
AST	UW	naïve	3	0.0002
		np	4	0.0002
		UW+H2	12	0.0002
	UW+H2	naïve	3	0.3181
		np	4	0.5835
		UW	6	0.0002
ALT	UW	naïve	3	0.0000
		np	4	0.0000
		UW+H2	12	0.0000
	UW+H2	naïve	3	0.1098
		np	4	0.1498
		UW	6	0.0000
LDH	UW	naïve	3	0.0002
		np	4	0.0001
		UW+H2	12	0.0003
	UW+H2	naïve	3	0.2917
		np	4	0.3154
		UW	6	0.0003
IRI score	UW	naïve	12	0.0000
		np	12	0.0000
		UW+H2	18	0.0002
	UW+H2	naïve	12	0.0001
		np	12	0.5893
		UW	18	0.0002
ASD	UW	naïve	3	0.0000
		np	3	0.1847
		UW+H2	3	0.0009
	UW+H2	naïve	3	0.0023
		np	3	0.0001
		UW	3	0.0009
ED1	UW	naïve	3	0.0003
		np	3	0.8731
		UW+H2	3	0.0025
	UW+H2	naïve	3	0.1180
		np	3	0.0089
		UW	3	0.0025

TUNEL	UW	naïve	3	0.0387
		np	3	0.6172
		UW+H2	3	0.7165
	UW+H2	naïve	3	0.1732
		np	3	0.1732
		UW	3	0.7165
CCL2	UW	naïve	3	0.1870
		np	4	0.2315
		UW+H2	5	0.8896
	UW+H2	naïve	3	0.4449
		np	4	0.0770
		UW	5	0.8896
ICAM1	UW	naïve	3	0.0148
		np	4	0.6505
		UW+H2	5	0.5709
	UW+H2	naïve	3	0.1127
		np	4	0.9999
		UW	5	0.5709
VCAM1	UW	naïve	3	0.0122
		np	3	0.0605
		UW+H2	3	0.5093
	UW+H2	naïve	3	0.0891
		np	3	0.4187
		UW	3	0.5093
HO1	UW	naïve	3	0.0387
		np	4	0.9245
		UW+H2	5	0.2652
	UW+H2	naïve	3	0.0019
		np	4	0.6394
		UW	5	0.2652
SIRT1	UW	naïve	3	0.4623
		np	3	0.0848
		UW+H2	3	0.3902
	UW+H2	naïve	3	0.0488
		np	3	0.6751
		UW	3	0.3902
HO1WB	UW	naïve	3	0.9985
		np	3	0.0122
		UW+H2	3	0.0000
	UW+H2	naïve	3	0.0000
		np	3	0.0000
		UW	3	0.0000
MDAserum	UW	naïve	3	0.8843
		np	3	0.3690
		UW+H2	3	0.0419
	UW+H2	naïve	3	0.1492
		np	3	0.6058
		UW	5	0.0419

MDAliver	UW	naïve	3	0.1109
		np	4	0.0311
		UW+H2	6	0.9964
	UW+H2	naïve	3	0.1510
		np	4	0.0436
		UW	6	0.9964
8OHdG	UW	naïve	3	0.0001
		np	3	0.0004
		UW+H2	4	0.2074
	UW+H2	naïve	3	0.0008
		np	3	0.0051
		UW	5	0.2074
4HNE	UW	naïve	3	0.0531
		np	3	0.1003
		UW+H2	3	0.1609
	UW+H2	naïve	3	0.8571
		np	3	0.0042
		UW	3	0.1609
